# Predominance of influenza B/Yamagata lineage viruses in Bulgaria during the 2017/2018 season

**DOI:** 10.1017/S0950268818003588

**Published:** 2019-01-25

**Authors:** N. S. Korsun, S. G. Angelova, I. T. Trifonova, I. L. Georgieva, I. S. Tzotcheva, S. D. Mileva, S. E. Voleva, A. M. Kurchatova, P. I. Perenovska

**Affiliations:** 1 Department of Virology, National Centre of Infectious and Parasitic Diseases (NCIPD), Sofia, Bulgaria; 2 Paediatric Clinic, University Hospital Alexandrovska, Sofia Medical University, Sofia, Bulgaria; 3 Department of Epidemiology, National Centre of Infectious and Parasitic Diseases (NCIPD), Sofia, Bulgaria

**Keywords:** Amino acid substitution, antigenic and genetic characterisation, influenza virus

## Abstract

In this study, we investigated the antigenic and genetic characteristics of influenza viruses circulating in Bulgaria during the 2017/2018 season. The detection and typing/subtyping of influenza viruses were performed using real-time RT-PCR. Results of antigenic characterisation, phylogenetic and amino acid sequence analyses of representative influenza strains are presented. The season was characterised by the predominance of B/Yamagata viruses, accounting for 77% of detected influenza viruses, followed by A(H1N1)pdm09 (17%), B/Victoria (3.7%) and A(H3N2) (2.4%). The sequenced B/Yamagata, B/Victoria, A(H1N1)pdm09 and A(H3N2) viruses belonged to the genetic groups 3, 1A, 6B.1 and 3C.2a1, respectively. Amino acid analysis of B/Yamagata isolates revealed the presence of three changes in haemagglutinin (HA), eight changes in neuraminidase (NA) and a number of substitutions in internal proteins compared with the B/Phucket/3073/2013 vaccine virus. Despite the amino acid changes, B/Yamagata viruses remained antigenically related to the vaccine strain. B/Victoria isolates fell into a group of viruses with double deletion (Δ162–163) in HA1. Substitutions in HA and NA sequences of B/Victoria, A(H1N1)pdm09 and A(H3N2) viruses were also identified compared with the vaccine strains, including in antigenic sites. The results of this study confirm the genetic variability of circulating influenza viruses and the need for continual antigenic and molecular surveillance.

## Introduction

Influenza is a highly contagious viral respiratory infection with the highest impact on population health in the EU/EEA compared with other infectious diseases due to its substantial incidence and associated mortality [1]. Annual influenza epidemics caused by influenza A and B viruses are estimated to result in about 3–5 million cases of severe illness and about 290 000–650 000 deaths, worldwide [2]. Influenza A viruses affect all age groups, have a large animal reservoir and are classified into subtypes based on the antigenic properties of their surface glycoproteins: haemagglutinin (HA) and neuraminidase (NA). Since 2009, two influenza A subtypes, A(H1N1)pdm09 and A(H3N2), together with influenza B viruses have been circulating worldwide, and they are included as antigens in influenza vaccines. Influenza B viruses are not categorised into subtypes but are divided into two phylogenetically and antigenically distinct lineages (Victoria and Yamagata), which have been co-circulating worldwide in variable proportions since the beginning of the XXI century [3–5]. These viruses are almost exclusively human pathogens, affect all age groups, but most commonly school-age children (aged 5–17 years) and in contrast to influenza A viruses do not pose a pandemic threat [6]. Recent studies have shown that clinical manifestations, influenza-associated complications and rate of hospitalisation of influenza B are similar to those of seasonal influenza A infection [7]. Influenza B viruses rarely represent over 50% of influenza cases, but when their activity is intense, they can cause severe epidemics [6, 8].

Influenza viruses are highly variable and undergo constant evolution, which proceeds by continuous replacement of genetic groups with new ones, leading to increases in the antigenic distances from the current vaccine strains and to a reduction of vaccine effectiveness. HA and NA are subjected to the strongest pressure by the host immune system resulting in amino acid changes and altered antigenicity. This process, known as ‘antigenic drift’, necessitates almost annual updating of the influenza vaccine composition in order to match the circulating strains. Previous studies have identified five major antigenic (antibody-recognizing) sites on the globular head of HA in the A(H1N1) subtype, designated as Sa, Sb, Ca 1/2, Cb, five (A-E) antigenic epitopes in the A(H3N2) subtype and four (120 loop, 150 loop, 160 loop, 190 helix) – in the type B [9–11]. A limited number of amino acid changes in the antigenic regions, especially at critical sites (in or near the receptor binding site, RBS) can create novel antigenic variants with unpredictable pathogenicity and with epidemiological significance [12]. The glycosylation of HA and NA is another adaptive viral mechanism to counteract the humoral immunity of the host. It has been supposed that the attached N-linked glycans physically shield antigenic epitopes, preventing immune recognition and protect the enzymatic sites of NA [13].

Phylogenetic and molecular analyses of influenza viruses are necessary to track their continuous evolution, to detect emergent antigenic variants, variants with increased transmissibility, virulence or variants with reduced sensitivity to antivirals. The main *aims* of this study are to investigate the circulation pattern of influenza viruses in Bulgaria during the 2017/2018 season, to determine their antigenic and genetic characteristics, to perform a molecular sequence analysis of the surface glycoproteins and internal proteins with the identification of amino-acid substitutions, compared with the vaccine and other reference strains.

## Materials and methods

### Influenza surveillance system

In Bulgaria, an acute respiratory infections (ARI) surveillance system is used to monitor influenza. It comprises a national sentinel network of general practitioners and paediatricians working in 218 outpatient health care facilities in all 28 major cities, regional centres and serving 381 493 people from all age groups (5.3% of the country population). During the period from November 1 to March 31, the primary care physicians report the daily number of new cases of ARI by age group, and between April and October, the data are reported on weekly basis (http://www.grippe.gateway.bg). Sentinel physicians take nose and throat swabs from a systematic selection of patients presenting with ARI and send them to the National Reference Laboratory (NRL) for influenza virus detection by real-time RT-PCR. It performs testing of clinical samples from the sentinel network and from severely ill patients hospitalised in different regions of the country. Overall positivity rates of sentinel specimens are used to estimate the start, the duration and the end of influenza activity; a 10% threshold is used to indicate the start of the seasonal epidemic (with at least 10 specimens tested). The peak of the season occurs when the positivity rate exceeds 50% [14].

### Study population and specimen collection

From week 40/2017 to week 20/2018, 1384 patients from different regions of Bulgaria treated for influenza-like illness or ARI in primary care settings or hospitals were enrolled in the National influenza surveillance programme. Combined nasal and throat specimens from the enrolled patients were collected with the help of commercial polyester collection swabs. Swabs were stored at 4 °C for up to 72 h before shipment to the laboratory. Specimens were processed immediately or stored at −80 °C before testing.

### Extraction of nucleic acids and real-time RT-PCR

Viral nucleic acids were extracted automatically from respiratory specimens using a commercial ExiPrep Dx Viral DNA/RNA kit (Bioneer, Korea) in accordance with the manufacturer's instructions. Detection and typing/subtyping of influenza viruses were carried out by a real-time RT-PCR method and the SuperScript III Platinum^®^ One-Step qRT-PCR System (Invitrogen, ThermoFisher Scientific, USA). All samples were first tested for the presence of influenza A and B viruses. The positive for influenza A samples were subsequently screened for A(H1N1)pdm09 and A(H3N2). The genetic lineage of detected influenza B viruses was also determined by real-time RT-PCR. Primers, probes and positive controls were provided by the International Reagent Resource (IRR), USA: CDC Influenza Virus Real-time RT-PCR A/B Typing Panel (FluRUO-01); A/H3/H1pdm09; Subtyping Panel (FluRUO-09); B lineage Genotyping Panel (FluRUO-05) and Influenza B/Victoria Lineage HA Gene Deletion Panel (FluRUO-10). Amplification was performed with a Chromo 4 thermal cycler (Bio-Rad) in accordance with the protocol of WHO (reverse transcription at 50 °C for 30 min, Taq inhibitor inactivation at 95 °C for 2 min, followed by 45 cycles of denaturation at 95 °C for 15 s and annealing/amplification at 55 °C for 30 s) [15, 16]. Samples with a cycle threshold (C_t_) value <38 were considered positive.

### Virus isolation and antigenic characterisation

All real-time RT-PCR-positive clinical specimens with C_t_ values <28 were inoculated into Madin Darby canine kidney (MDCK) and MDCK-SIAT1 (that express increased levels of *α*2,6-sialyl transferase) [17] cell cultures. Cultures were incubated at 35 °C in a 5% CO_2_ atmosphere and observed on a daily basis for 7 days for evidence of cytopathology. The presence of a virus in a culture was confirmed by haemagglutination assay following standard protocols using a 1% suspension of guinea pig red blood cells. The antigenicity of Bulgarian isolates was characterised using the haemagglutination inhibition (HI) assay with panels of reference viruses and antisera at the WHO Collaborating Centres (WHO-CC) in London and Atlanta. The HI titre was determined as the reciprocal of the highest dilution of antiserum that completely inhibits virus-induced haemagglutination [15]. Viral isolates were identified as antigenically related to the vaccine virus if they showed no more than fourfold reduced HI titre with antiserum raised against the vaccine virus, as compared with the homologous titre. A reduction of at least eightfold in the HI titres was considered a signal of antigenic drift.

### Genetic characterisation

Clinical specimens, positive for influenza viruses and virus isolates, were sequenced using the Sanger method at the WHO-CC, London, and using Next Generation Sequencing (NGS) at the WHO-CC, Atlanta. Sequences were deposited in the Global Initiative on Sharing All Influenza Data (GISAID) (http://www.gisaid.org) [18] under the isolates ID: for *B/Yamagata*: EPI_ISL_ 296666; EPI_ISL_ 296667; EPI_ISL_ 296811; EPI_ISL_ 308677; EPI_ISL_ 308678; EPI_ISL_ 314121–314127; EPI_ISL_ 314129–314132; for *B/Victoria*: EPI_ISL_ 314133; EPI_ISL_ 314134; for *A(H1N1)pdm09*: EPI_ISL_ 299022; EPI_ISL_ 299023; EPI_ISL_ 308418; EPI_ISL_ 311872–311885; EPI_ISL_ 314190–314193; EPI_ISL_ 316679; for *A(H3N2)*: EPI_ISL_ 31260; EPI_ISL_ 312261. For the purposes of phylogenetic analyses, the study sequences, together with sequences of reference viruses whose genetic group identities were known and of viruses circulating in different countries of Europe during the 2017/2018 season, were retrieved from the GISAID. They were all aligned using the MUSCLE program embedded in the Molecular Evolutionary Genetics Analysis (MEGA, version 6.06; http://www.megasoftware.net/) software [19]. Best nucleotide substitution models for phylogenetic analysis of HA (Hasegawa–Kishino–Yano model with a *γ* distribution, HKY+G) and NA (Tamura 3-parameter model with a *γ* distribution, T92+G) were determined using MEGA 6.06. Phylogenetic trees were constructed using the Maximum Likelihood method within the MEGA 6.06. The reliability of the tree topology was assessed by bootstrapping with 1000 replications.

### Deduced amino acid sequence analysis and prediction of N-glycosylation motifs

The amino acid sequences were generated by translating nucleotide sequences with the standard genetic code using the MEGA software. The deduced amino acid sequences of the study strains were compared with those of vaccine strains and other reference strains to identify amino acid substitutions. The amino acid identity was calculated using FluSurver (http://flusurver.bii.a-star.edu.sg). The potential N-linked glycosylation sites (NGS) in the HA and NA were predicted using the NetNGlyc 1.0 web Server (http://www.cbs.dtu.dk/services/NetNGlyc) to identify sequence motifs N–*X*–S/T (sequon), where *X* can be any amino acid except proline.

### Antiviral susceptibility testing

The screening of A(H1N1)pdm09 viruses for the presence of point mutation conferring H275Y oseltamivir resistance was carried out using a real-time RT-PCR assay that allowed discrimination of a single nucleotide difference between oseltamivir-sensitive and resistant viruses. Primer/probe sequences and protocol were kindly provided by Public Health England, London.

## Results

In the 2017/2018 season, the influenza epidemic in Bulgaria started in week 1/2018 and continued until week 9/2018. The epidemic peak was reached in the week 4/2018 with 8806 ARI cases registered and 230.83 per 10 000 population incidence rate. The highest consultation rate for ARI was observed in the 5–14 years age group, followed by the 0–4 years age group with maximum values of 722.84 and 719.84 per 10 000 population, respectively (http://www.grippe.gateway.bg).

### Influenza virus detection

**Fig. 1.**
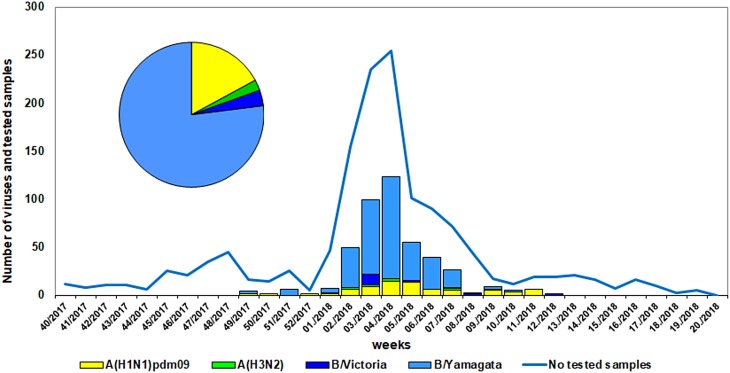
Weekly distribution of influenza virus detections during the 2017/2018 season.

Influenza viruses were detected in 457 (33%) of 1384 patient samples screened. Of these, 89 (19.5%) were positive for influenza type A virus and 368 (80.5%) for type B. Among the influenza A viruses, 78 (87.6%) were A(H1N1)pdm09 and 11 (12.4%) – A(H3N2); among the type B viruses, 352 (95.7%) belonged to the Yamagata-lineage and 16 (4.3%) to the Victoria-lineage (Fig. 1). Further testing using a real-time RT-PCR Influenza B/Victoria Lineage HA Gene Deletion Panel indicated that all 16 B/Victoria viruses belong to the newly emerged variant with double deletion (Δ162–163) in HA1. Of the patients with influenza B/Yamagata virus infection, 99 (28.1%) were outpatients and 253 (71.9%) – hospitalised. Three B/Yamagata virus-infected individuals were admitted to intensive care units and one of them aged 71 years died during hospitalisation.

The first three influenza viruses of the season (B/Yamagata) were identified in week 49/2017 and the peak of influenza virus detections was reached in week 4/2018. The last influenza virus (B/Yamagata) was detected in week 12/2018.

### Age distribution

**Fig. 2.**
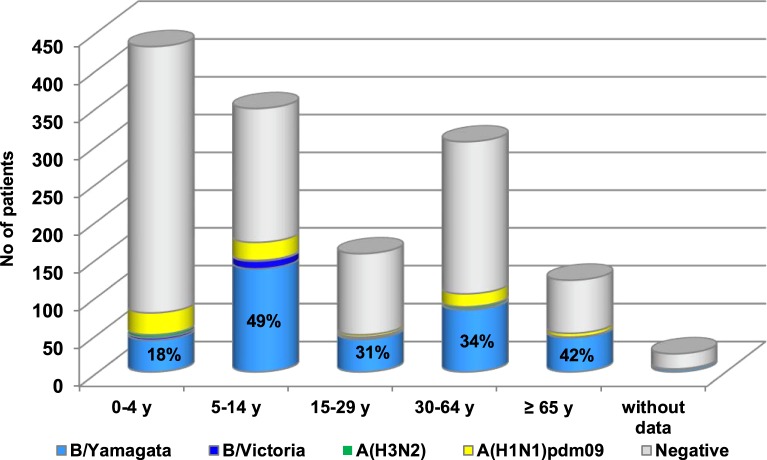
Age distribution of patients with detected influenza viruses during the 2017/2018 season. The values indicate the percentages of patients positive for all influenza viruses in the individual age groups.

Influenza viruses were detected in all studied age groups (Fig. 2). The largest proportion of influenza-positive patients was found in the age group 5–14 years (49%), followed by the age group ⩾65 years (42%). B/Yamagata-lineage viruses, which were predominant in the 2017/2018 season, caused illness most frequently in the age groups of 5–14 (39.3%) and ⩾65 year olds (37.2%).

### Virus antigenic characterisation

In early January 2018, the first 19 detected influenza viruses (clinical specimens) were sent to the WHO-CCs in London and Atlanta for further characterisation. In the course of the 2017/2018 season, a total of 52 influenza viruses were isolated on MDCK-SIAT1 cell culture. Thirty-nine representative influenza isolates were sent to the WHO-CC, London, where they were characterised in detail.

A total of 16 B/Yamagata-lineage viruses was antigenically characterised by HI assay using post-infection ferret antisera raised against reference viruses. The test viruses showed 2-4-fold reduced reactivity with antisera raised against clade 3 viruses, including the antiserum raised against B/Phuket/3073/2013 strain, the component of the 2017/2018 Northern Hemisphere (NH) quadrivalent influenza vaccine [20]. The study isolates were recognised less well by antisera raised against viruses in clade 2, the B/Massachusetts/02/2012 clade. Two B/Victoria-lineage isolates reacted poorly to the antiserum against current vaccine virus, egg-propagated B/Brisbane/60/2008, but they were inhibited well by the antisera raised against viruses with a deletion of two amino acids (Δ162–163) in HA1 – B/Norway/2409/2017 and B/Colorado/06/2017, the recommended vaccine strain for use in the 2018–2019 NH influenza season [21].

Twenty-two A(H1N1)pdm09 isolates analysed were well inhibited by the panel of antisera including the antiserum raised against A/Michigan/45/2015 strain, the A(H1N1)pdm09 component of the 2017/2018 and 2018/2019 NH vaccines, indicating antigenic similarity to the vaccine strain. Two A(H3N2) test viruses showed 2–4-fold reduced reactivity with antiserum raised against egg-propagated A/Singapore/INFIMH-16-0019/2016, the recommended vaccine component for use in the 2018–2019 NH influenza season. In contrast, both viruses reacted poorly to the antiserum against current vaccine virus, egg-propagated A/Hong Kong/4801/2014.

### Phylogenetic analysis

**Fig. 3.**
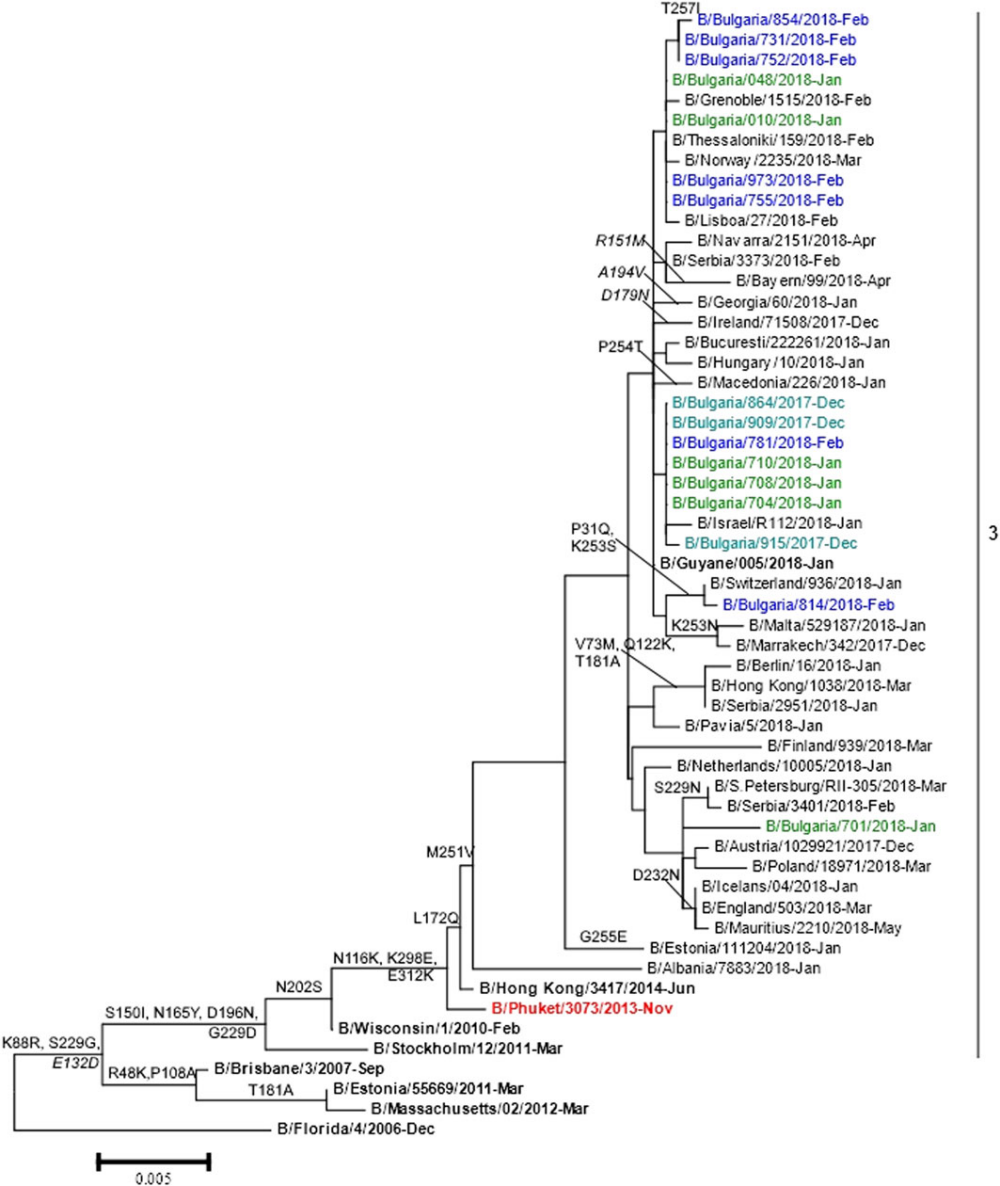
HA gene phylogeny of influenza B/Yamagata viruses detected in Bulgaria during the 2017/2018 season. Reference viruses are indicated in bold and vaccine virus B/Phuket/3073/2013 in red. Bulgarian viruses detected in December 2017, January and February 2018 are indicated in teal, green and blue, respectively. Substitutions in the HA2 subunit are indicated in italic.

Phylogenetic trees based on HA and NA genes were constructed. All 16 B/Yamagata-lineage viruses analysed belonged to phylogenetic clade 3, the B/Wisconsin/1/2010 – B/Phuket/3073/2013 clade (Fig. 3). Two B/Victoria-lineage viruses sequenced fell into genetic clade 1A. They clustered together with viruses with a deletion in positions 162–163 of HA1 represented by the B/Colorado/06/2017 vaccine strain (data not shown).

**Fig. 4.**
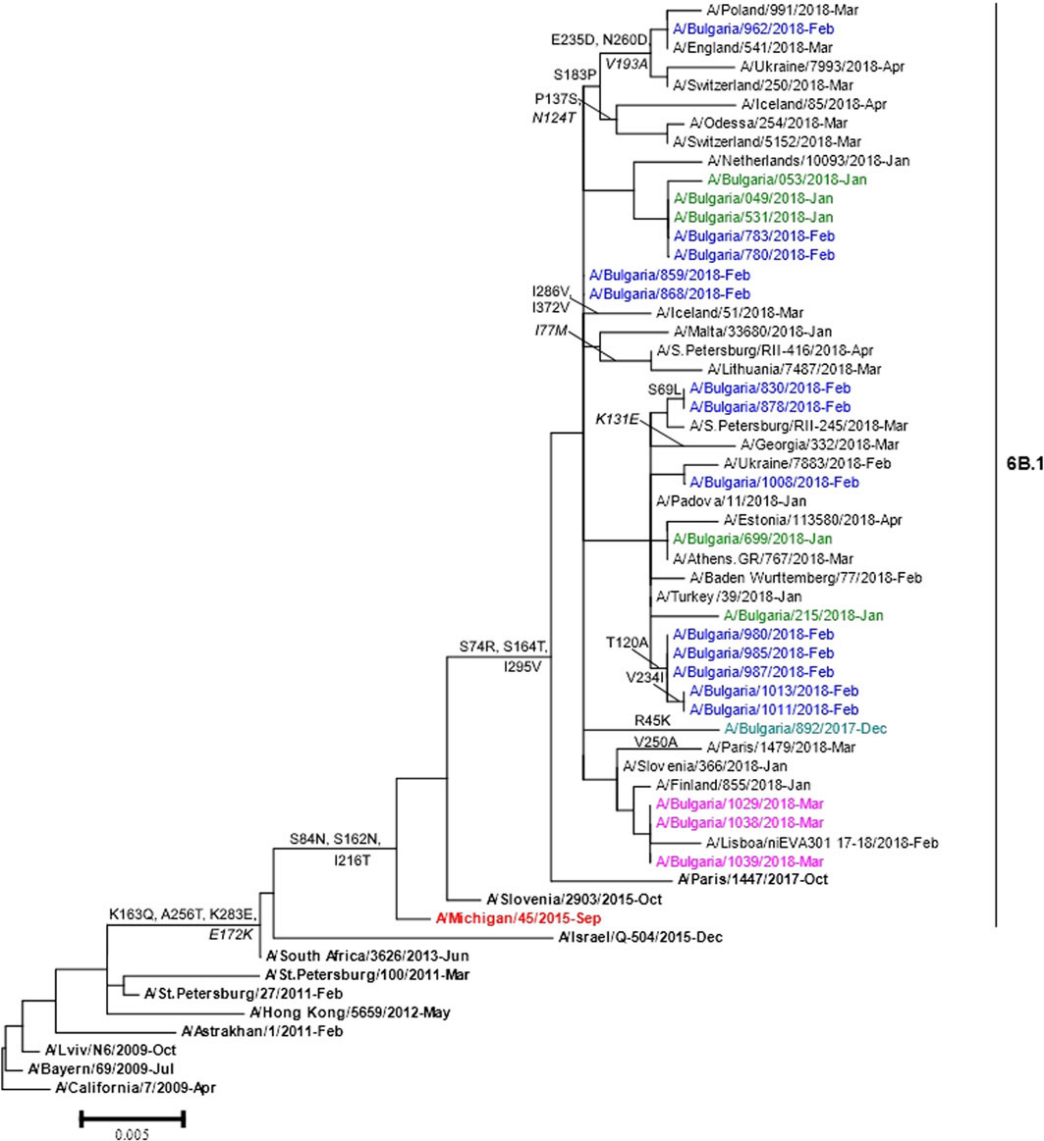
HA gene phylogeny of influenza A(H1N1)pdm09 viruses detected in Bulgaria during the 2017/2018 season. Reference viruses are indicated in bold and vaccine virus A/Michigan/45/2015 in red. Bulgarian viruses detected in December 2017, January, February and March 2018 are indicated in teal, green, blue and pink, respectively. Substitutions in the HA2 subunit are indicated in italic.

Phylogenetic analysis of 22 A(H1N1)pdm09 isolates showed that they belonged to subclade 6B.1 represented by globally circulating A(H1N1)pdm09 viruses and clustered with the A/Michigan/45/2015 vaccine strain (Fig. 4). Two Bulgarian A(H3N2) isolates fell into genetic subclade 3C.2a1 and clustered with A/Singapore/INFIMH-16-0019/2016 (data not shown).

### Amino acid sequence analysis

The complete HA and NA amino acid sequences of the Bulgarian influenza viruses were compared with those of corresponding vaccine strains in order to identify substitutions that could change the antigenic properties of viruses and may have had an impact on vaccine effеctiveness.

#### B/Yamagata

Genetic analysis of Bulgarian B/Yamagata isolates indicated that the HA amino acid sequences had a high degree of similarity (amino acid identity of 99.1–99.5%) to that of the vaccine strain, B/Phuket/3073/2013. Amino acid analysis revealed the presence of three amino acid substitutions in HA with respect to the vaccine virus: L172Q, D196N (leading to the formation of a new NGS in the antigenic site, 190-helix) and M251V. The NA sequences contained changes in eight positions as compared with the B/Phuket/3073/2013 vaccine strain: I49M, C54Y, R65H, I171M, P244S, D342K, K373Q and S402P. The catalytic pocket and framework sites supporting the catalytic residues in NA were conserved among all 16 viruses studied [22]. Bulgarian isolates possessed 11 NGS in HA (HA1 positions 25, 59, 145, 167, 196, 303 and 332, and HA2 positions 145, 171, 184 and 216) and five in NA (positions 56, 64, 144, 284 and 463). A variation NRS→NHS in position 65 at the NA glycosylation site was identified in all but one study isolates. NGS at positions 145, 167 and 196 fell in antigenic sites 150-loop, 160-loop and 190-helix, respectively [11].

The amino acid sequences of the internal proteins of two Bulgarian B/Yamagata viruses were compared with those of the B/Phuket/3073/2013 vaccine strain. The study sequences possessed the following amino acid substitutions: Q354H in PB2; A591S in PB1; M326V, S547G and I594V in PA; T313A in NP; M21V in M2; K110E, P114L and K176R in NS1. No substitutions were found in the proteins M1 and NEP.

#### B/Victoria

The HA and NA amino acid sequences of two Bulgarian isolates contained changes in four and seven positions, respectively, compared with the B/Brisbane/60/2008 vaccine strain. Two substitutions in HA (I117V and N129G) were located in the antigenic site, 120-loop. Both Bulgarian isolates fell into a group of viruses that have a six-nucleotide deletion in the HA gene segment (encoding amino acids 162 and 163) and share the substitutions N129G, I180V in HA1, R151K in HA2 and K371Q in the NA. The study viruses possessed the same 12 conserved NGS in HA (HA1 positions 25, 59, 145, 166, 197, 233, 304 and 333, and HA2 positions 145, 171, 184 and 216) and four in NA (positions 56, 64, 144 and 284) identified in B/Brisbane/60/2008. Three NGS (HA1 positions 145, 166 and 197) were located in the antigenic epitopes 150-loop, 160-loop and 190-helix, respectively.

#### A(H1N1)pdm09

The sequence data of the Bulgarian isolates showed a high homology for HA protein to that of the vaccine strain, A/Michigan/45/2015 (amino acid identity of 98.4–99.1%). Changes in four positions of HA and 12 positions of NA were identified when they were compared with the sequences of the vaccine strain. The HA protein of Bulgarian strain A/Bulgaria/962/2018 fell into a group of viruses that shared the substitutions S183P, E235D and N260D in HA1 and V193A in HA2. Eleven other Bulgarian strains shared the substitution T120A in HA1 with a large number of viruses. The substitutions S74R and S164T were located in antigenic sites Cb and Sa, respectively. The isolate A/Bulgaria/962/2018 possessed additional substitution E235D located in antigenic site Ca [23]. All study isolates carried eight potential NGS in HA (HA1 positions 10, 23, 87, 162, 276, 287 and HA2 positions 154 and 213) and eight in NA (positions 42, 50, 58, 63, 68, 88, 146 and 235), as the vaccine strain. Each one of the examined strains possessed the variation NQS→NQT in position 162 at the HA1 glycosylation site.

#### A(H3N2)

Comparison of the amino acid sequences of HA protein of two Bulgarian A(H3N2) isolates with the corresponding A/Hong Kong/4801/2014 vaccine strain revealed changes at 10 positions, six of which were unusual: Q75H, R142G, N171R, I192N, M320I in HA1 and A201V in HA2. The substitutions R142G, T160K and Q75H were located in antigenic sites A, B and E, respectively. The substitution T160K, possibly associated with propagation in cell culture, resulted in a loss of NGS. Twelve potential NGS in HA (HA1 positions 8, 22, 38, 45, 63, 122, 126, 133, 165, 246 and 285, and HA2 position 154) were identified. The mutation G155E in HA2 altered the potential glycosylation motif from NGT to NET. The only Bulgarian NA sequence analysed, differed from the NA of the A/Hong Kong/4801/2014 vaccine strain by 11 amino acid substitutions. The substitution N86D resulted in a loss of NGS and the substitution S245N resulted in a formation of a new NGS. Eight potential NGS in NA (61, 70, 146, 200, 234, 245, 329 and 367) were identified, with two (146 and 367) being located around the enzymatic active site [24].

#### Antiviral susceptibility testing

All 78 detected A(H1N1)pdm09 viruses were analysed by real-time RT-PCR with respect to the H275Y mutation in the NA sequence as a marker of the resistance to oseltamivir, none of the isolates harboured this substitution. The Bulgarian isolates did not contain other known markers of resistance or reduced susceptibility to neuraminidase inhibitors in the NA gene segment [25].

## Discussion

In Bulgaria, the 2017/2018 influenza epidemic was characterised by an unexpectedly strong prevalence of B/Yamagata-lineage viruses, accounting for 77% of all positive influenza cases and a low circulation of B/Victoria-lineage (3.5%) and A(H3N2) (2.4%) viruses. The similar predominance of B/Yamagata viruses was observed in the most European countries [26]. In Bulgaria, the proportion of influenza B viruses identified during the eight seasons after the 2009/2010 pandemic ranged between 1% and 77% (on average 31%) of all influenza virus detections. This average circulation frequency was higher in comparison with that reported by Global influenza B study (22.6%) for the 2000–2013 period [6]. The intensive circulation of type B viruses was also observed in Bulgaria during the 2012/2013 season [27]. In agreement with other studies, children aged 5–14 years were most affected but a significant B/Yamagata virus-positive rate was also found in subjects ⩾65 years of age [28, 29].

Previous observations exhibit that influenza B viruses are genetically more stable, evolve more slowly and undergo antigenic drift less rapidly as compared with type A viruses [30, 31]. The reassortment of gene segments between and within lineages, as well as different deletions, insertions and substitutions, generate the genetic diversity of these viruses and allow them to escape host immunity [32–34].

A limited number of amino acid changes were identified in HA, NA and internal proteins of the study B/Yamagata viruses with respect to the B/Phuket/3073/2013 vaccine strain that has been in use since 2015. The antigenic regions – 120-loop (positions 116–137), 150-loop (positions 141–150) and 160-loop (positions 162–167) were identical to those in vaccine strain. One amino acid substitution and one new NGS were identified in the fourth antigenic region, 190-helix (positions 194–202) which forms part of the RBS. Amino acid variations in the internal proteins have also been found by other authors, but the biological significance of thesе substitutions has yet to be precisely elucidated [21, 35]. Despite amino acid/NGS changes the studied Bulgarian viruses preserved the antigenic specificity of B/Phuket/3073/2013-like strains. The unusually intensive spread of Yamagata-lineage viruses in the 2017/2018 season can be explained by their low circulation over the previous two seasons, and not by antigenic drift.

As regards B/Victoria isolates, more amino acid variations were identified compared with the corresponding B/Brisbane/60/2008 vaccine virus which resulted in antigenic changes. Two substitutions were located in antigenic 120-loop which is one of the most frequently changing regions [11, 36]. The attachment of the oligosaccharide chains to antigenic sites probably facilitates immune evasion. All 16 Bulgarian B/Victoria viruses fell into a big group of viruses with double deletion (Δ162–163) in the antigenic 160-loop of HA. Viruses carrying this double deletion were first reported in the USA (in Europe – in Norway) during the 2016/2017 season and were antigenically distinct from B/Brisbane/60/2008 [20].

All Bulgarian A(H1N1)pdm09 viruses were genetically and antigenically very similar to the A/Michigan/45/2015 vaccine strain used for the first time this season as well as to the vast majority of circulating A(H1N1)pdm09 viruses worldwide. Only four amino acid substitutions in HA were identified, compared with the A/Michigan/45/2015 vaccine strain, two of them were located in antigenic regions.

In Europe, influenza A(H3N2) viruses belonging to clade 3C.2a emerged at the end of the 2013/2014 season and since the 2014/2015 season has been the predominant clade. Over this period they underwent considerable genetic diversification and diverged in several subclades and subgroups: subclade 3C.2a1 (subgroups 3C.2a1a and 3C.2a1b), subclades 3C.2a2, 3C.2a3 and 3C.2a4, defined by certain amino acid substitutions [25, 37]. In the 2017/2018 season in Bulgaria, influenza A(H3N2) viruses had the lowest incidence rate (0.8%) among circulating influenza viruses and the only two sequenced isolates fell within subclade 3C.2a1. Significant changes (10 substitutions in HA and 11 in NA) were identified compared with the actual A/Hong Kong/4801/2014 vaccine strain, including two amino acid substitutions in antigenic sites A and B located on the top of HA around the RBS and one substitution in antigenic site E [12]. The substantially greater mutation rate and the higher degree of glycosylation of A/H3 viruses are at the basis of their greater variability and evolutionary plasticity compared with the other seasonal influenza viruses [38].

## Conclusion

The 2017/2018 season was unique with the exclusive high activity of influenza B viruses, which occurs once every 5–10 years [6, 35, 39, 40]. The predominant circulating Yamagata lineage was not included in the trivalent influenza vaccine, which suggests that the 2017/2018 trivalent vaccines provided suboptimal protection against prevailing influenza B viruses due to the low level of cross-protection between both B lineages. In contrast, the use of a quadrivalent vaccine allowеd the overcome of the mismatch between vaccine and epidemic B viruses. The results of this study indicate the need for continual antigenic and molecular surveillance of circulating influenza viruses for the purpose of early detection of novel genetic variants of public health and clinical significance.
